# Supplementary material for: Ancient and Recent Riverine Gene Flow Contributed to the Adaptive Radiation of Sailfin Silversides in Wallace's Dreampond
Source: Mol Ecol. 2026 Jun 10;35(11):e70414. doi: 10.1111/mec.70414 (PMC13253165; doi:10.1111/mec.70414)

**Ancient and recent riverine gene flow contributed to the adaptive radiation of sailfin silversides in Wallace's Dreampond**

Short title: Gene flow in adaptive radiation of sailfin silversides

Els Lea R De Keyzer^1,2^, Fabian Herder^3^, Astrid Böhne^3^, Francisco Campuzano Jiménez^1^, Valentina Burskaia^1^, Sandra Kukowka^3^, Alan Tracey^4^, Amy Denton^4^, Graeme Oatley^4^, Wellcome Sanger Institute Tree of Life programme^4 ,5^, Wellcome Sanger Institute Scientific Operations: DNA Pipelines collective^4,6^, Tree of Life Core Informatics collective^4,7^, Daniel F Mokodongan^8^, Daisy Wowor^8^, Hannes Svardal^1 ,9^

^1^ Evolutionary Ecology Group, Department of Biology, University of Antwerp, 2020 Antwerp, Belgium

^2^ Aquatic Macrofauna Biodiversity and Conservation Research Group, Hasanuddin University, Makassar 90245, South Sulawesi, Indonesia.

^3^ Leibniz-Institute for the Analysis of Biodiversity Change (LIB), Museum Koenig Bonn, 53113 Bonn, Germany

^4^ Wellcome Sanger Institute, Hinxton, Cambridgeshire CB10 1SA, UK

^5^ members: https://doi.org/10.5281/zenodo.4783585

^6^ members: https://doi.org/10.5281/zenodo.4790455

^7^ members: https://doi.org/10.5281/zenodo.7116866

^8^ Museum Zoologicum Bogoriense, Research Center for Biosystematics and Evolution, National Research and Innovation Agency (BRIN), Cibinong, 16911, Indonesia

^9^ Naturalis Biodiversity Center, 2333 Leiden, The Netherlands

Corresponding authors: Els L. R. De Keyzer, Hannes Svardal

Email: els.dekeyzer@uantwerpen.be, hannes.svardal@uantwerpen.be

Supplementary figures


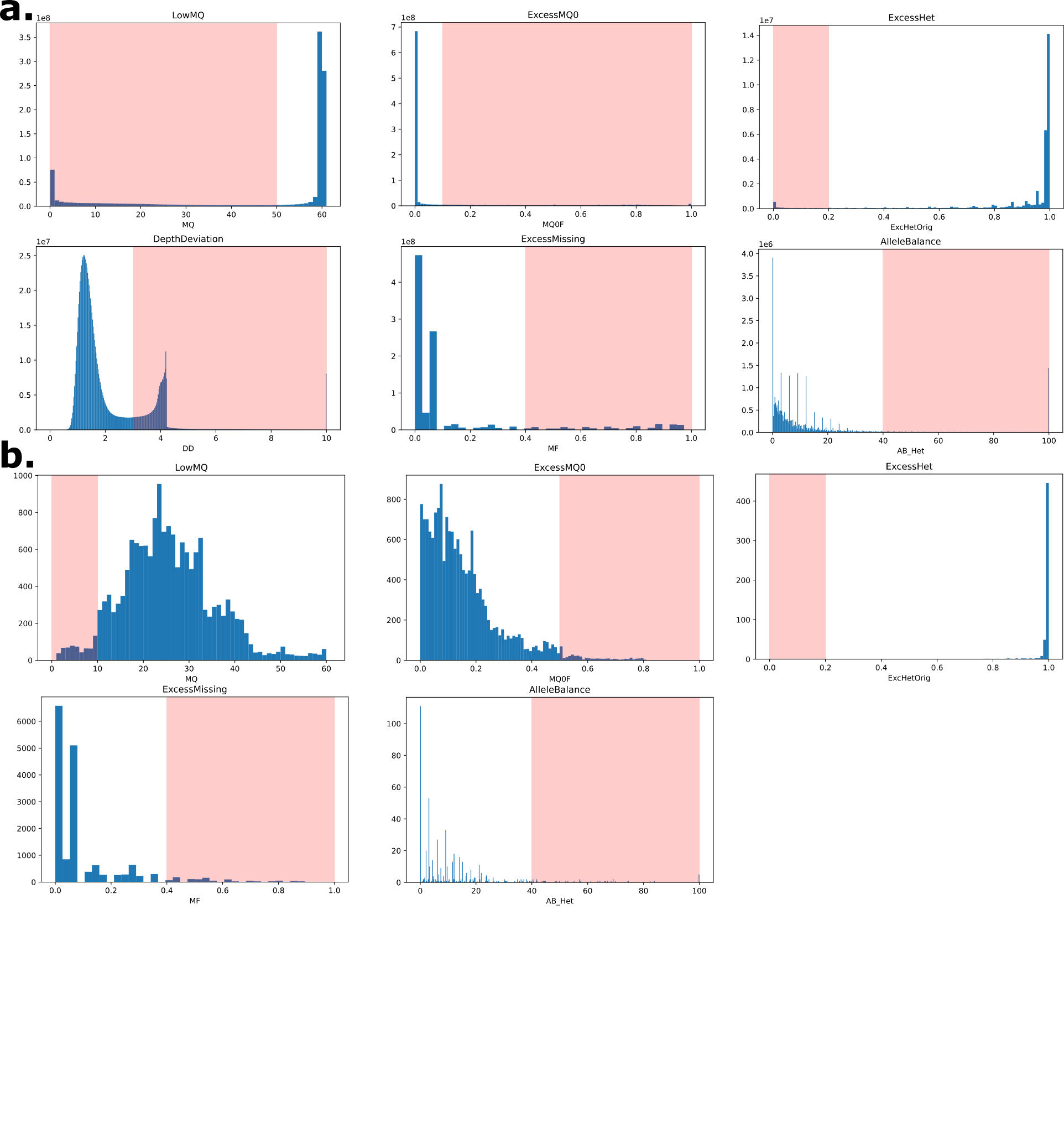


[Supplementary figure S1](#figur_filter). **Filter settings and number of sequences removed per filter**. Area highlighted in red indicates the filter threshold (a) for the nuclear genome and (b) for the mitochondrial DNA.


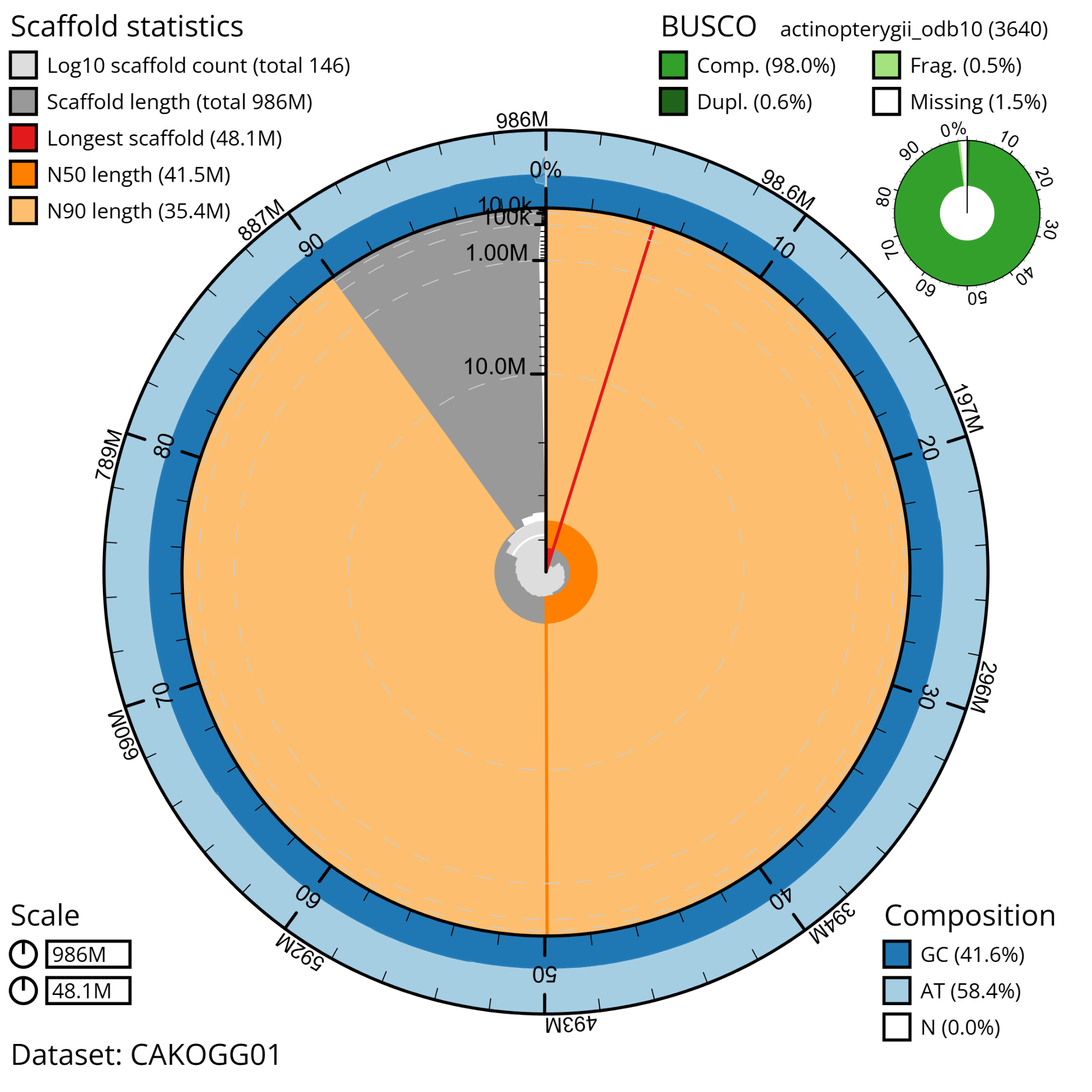


## [Supplementary figure S2](#figur_refsnail). Assembly statistics for reference genome assembly of *Telmatherina bonti*. The BlobToolKit Snailplot shows N50 metrics and BUSCO gene completeness. The main plot is divided into 1,000 size-ordered bins around the circumference with each bin representing 0.1% of the 986,039,709 bp assembly. The distribution of sequence lengths is shown in dark grey (length measured from outside to inside) with the plot radius scaled to the longest sequence present in the assembly shown in red (48,058,054 bp). Orange and pale-orange arcs show the N50 and N90 sequence lengths (41,523,244 and 35,388,860 bp), respectively. The pale grey spiral shows the cumulative sequence count on a log scale with white scale lines showing successive orders of magnitude. The blue and pale-blue area around the outside of the plot shows the distribution of GC, AT and N percentages in the same bins as the inner plot. Top right: summary of complete, fragmented, duplicated and missing BUSCO genes in the actinopterygii_odb10 set. An interactive version of this figure is available at https://blobtoolkit.genomehubs.org/view/Telmatherina%20bonti/dataset/CAKOGG01/snail.


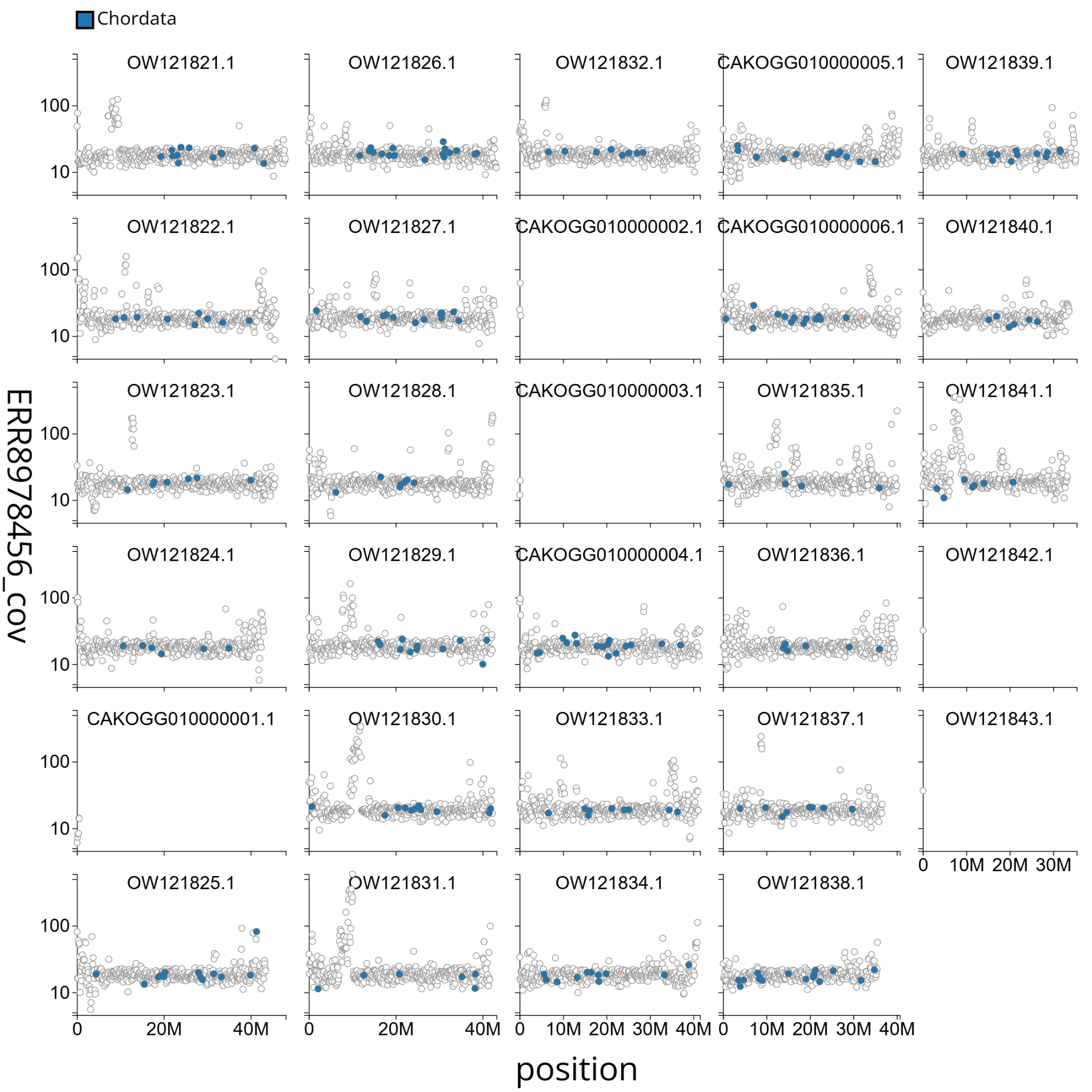


[Supplementary figure S3](#figur_refcov)**. Distribution plot of base coverage in ERR8978456 against position for sequences in assembly CAKOGG01.** 100kb windows are coloured by phylum.

**
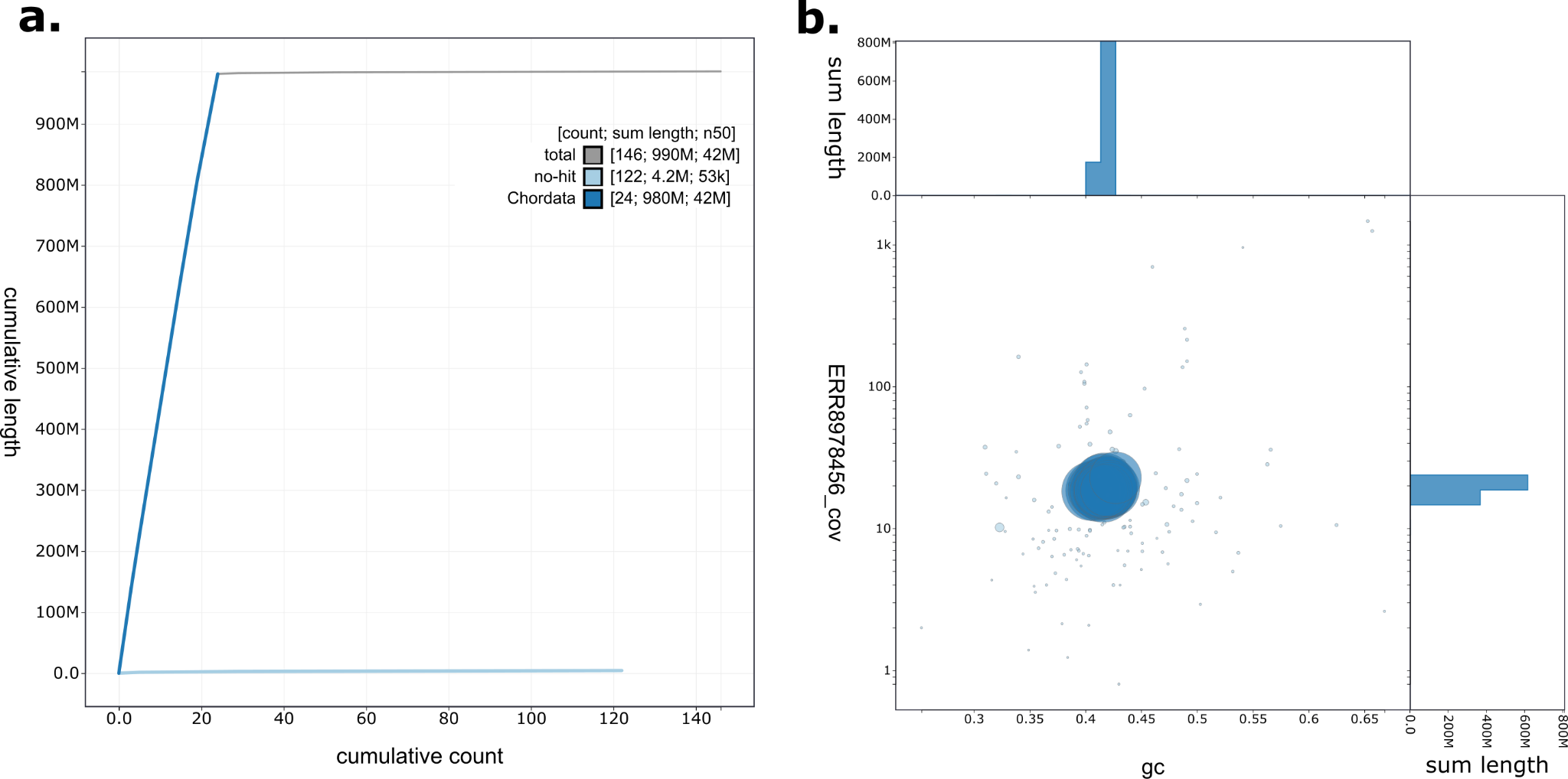
**

S[upplementary figure S4](#figur_refcomp)**. a. Cumulative sequence length for assembly CAKOGG01.** The grey line shows cumulative length for all sequences. Coloured lines show cumulative lengths of sequences assigned to each phylum using the buscogenes taxrules. An interactive version of this figure is available at https://blobtoolkit.genomehubs.org/view/Telmatherina%20bonti/dataset/CAKOGG01/cumulative **b. Blob plot of base coverage in ERR8978456 against GC proportion for sequences in assembly CAKOGG01.** Sequences are coloured by phylum. Circles are sized in proportion to sequence length. Histograms show the distribution of sequence length sum along each axis. An inveractive version of this figure is available at https://blobtoolkit.genomehubs.org/view/Telmatherina%20bonti/dataset/CAKOGG01/blob


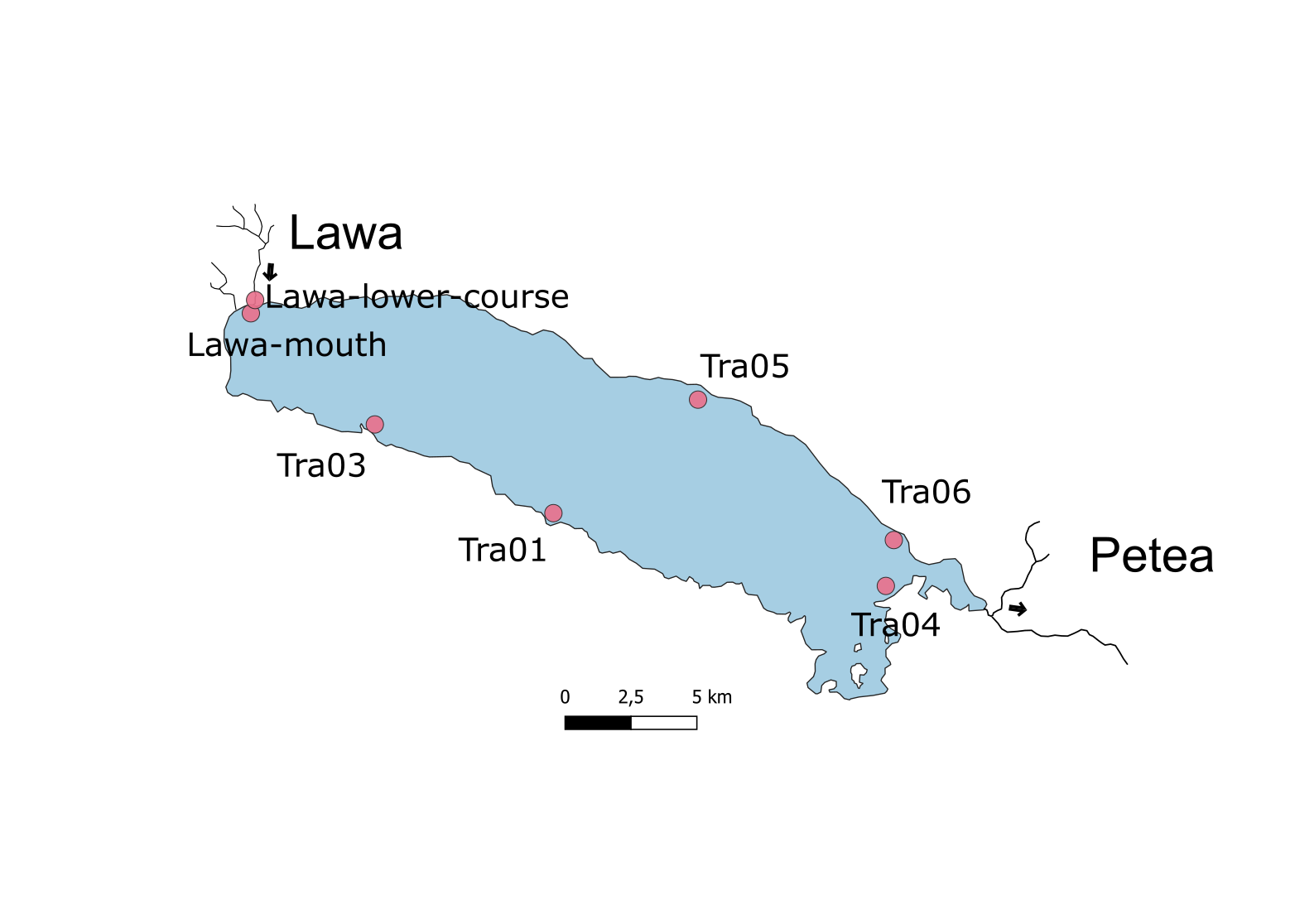


[Supplementary figure S5](#figur_locations). **Map of sampling locations.** Names of sampling locations correspond to sample information as displayed in supplementary table S2. Inflow river Lawa and outflow river Petea are indicated.


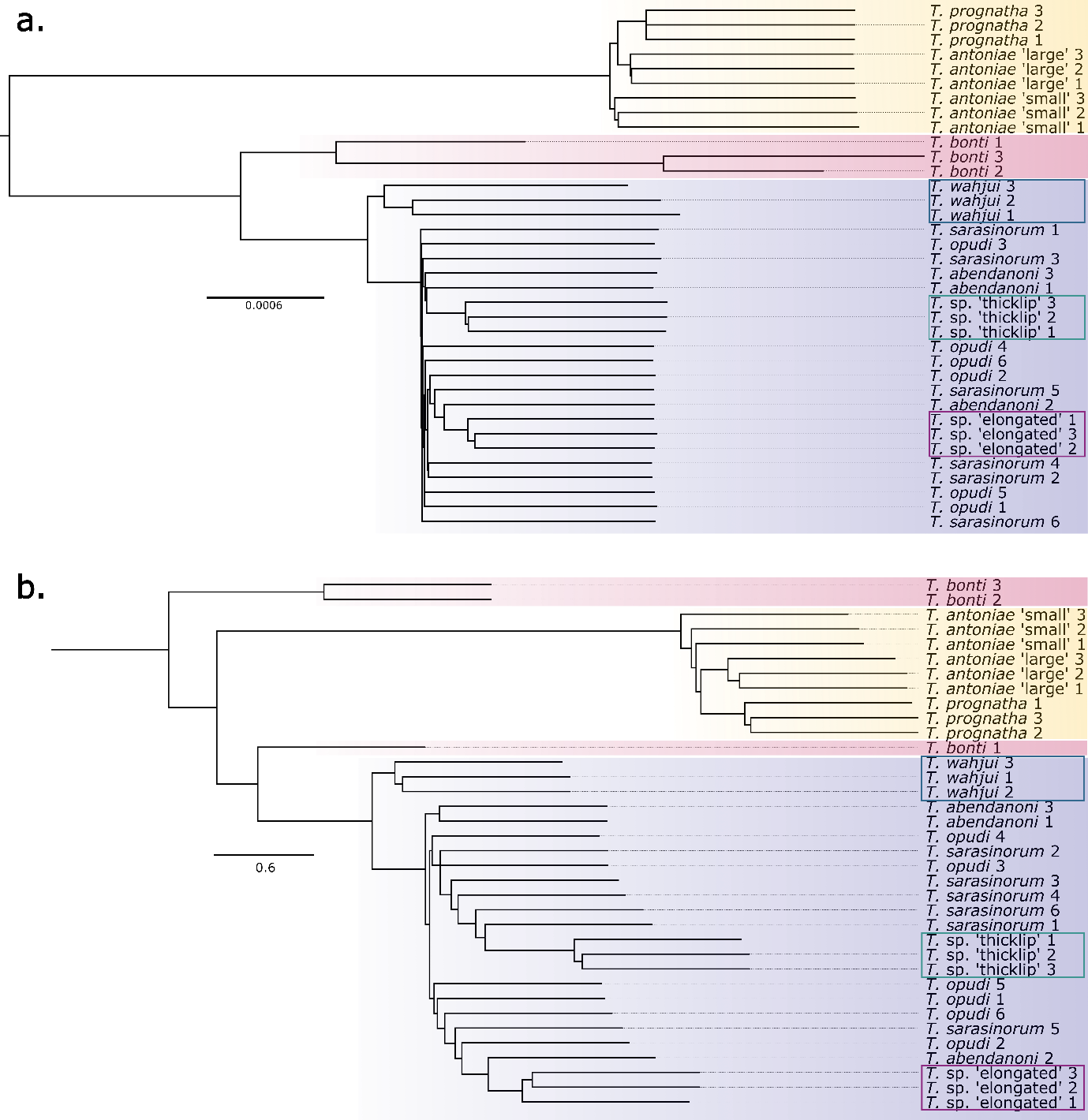


[Supplementary figure S6](#figur_NJtree). a. **NJ tree based on distance matrix**. Scale is the number of mutations divided by accessible genome size b. **astral consensus of NJ** **trees** based local distance trees based on 100 kb windows. All trees are rooted against a sample of the outgroup species *M. ladigesi*. Sharpfin species are highlighted in purple, roundfins in yellow and the *T. bonti* samples are indicated in red background shading.


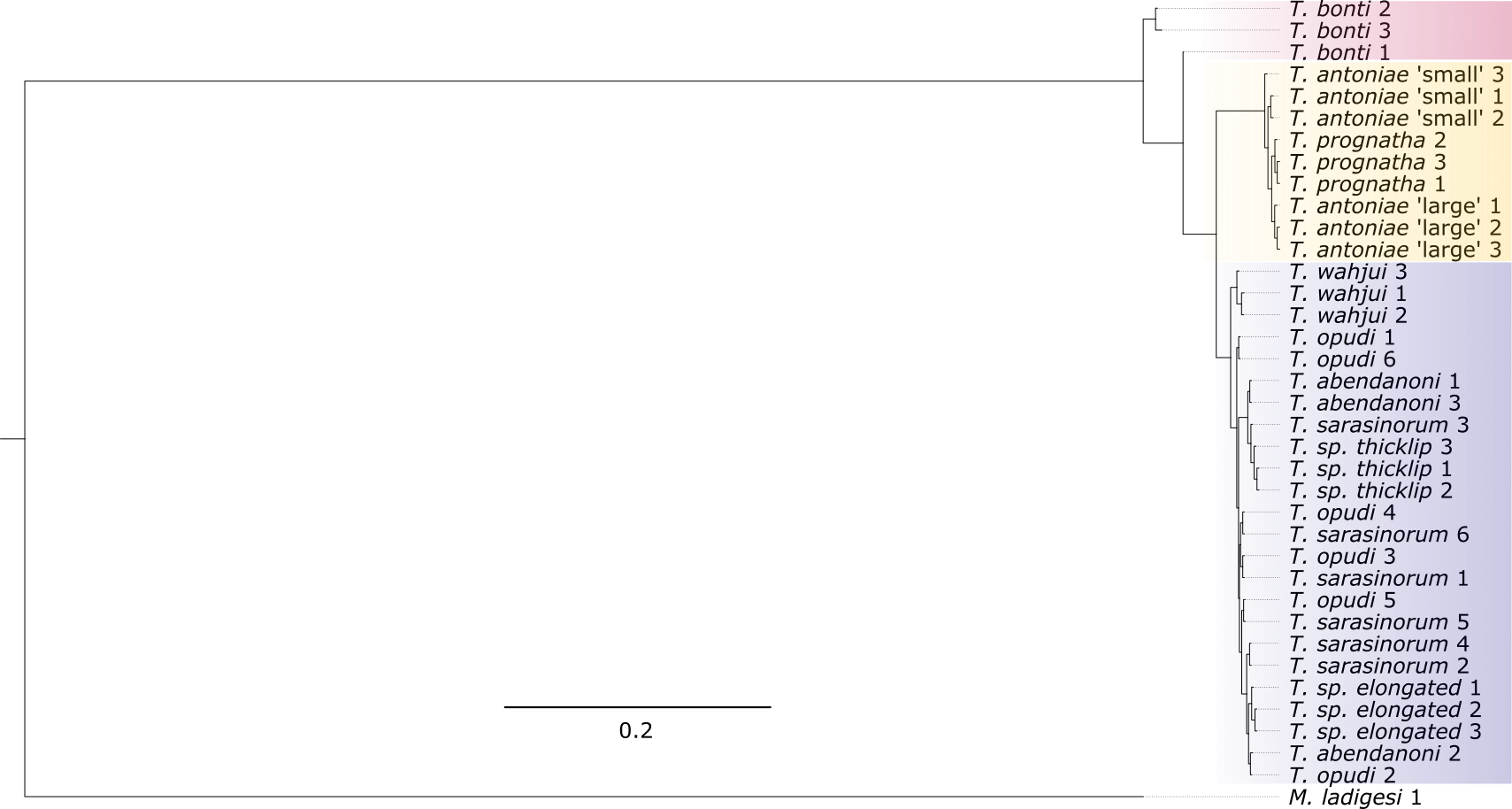


[Supplementary figure S7](#figur_outgroup). **Phylogeny based on maximum likelihood (ML) trees** with outgroup species *Marosatherina ladigesi*. Consensus phylogeny based on 9150 ML trees in 100 kb windows across the genome. Sharpfin species are highlighted in purple, roundfins in yellow and the *T. bonti* samples are indicated in red background shading.


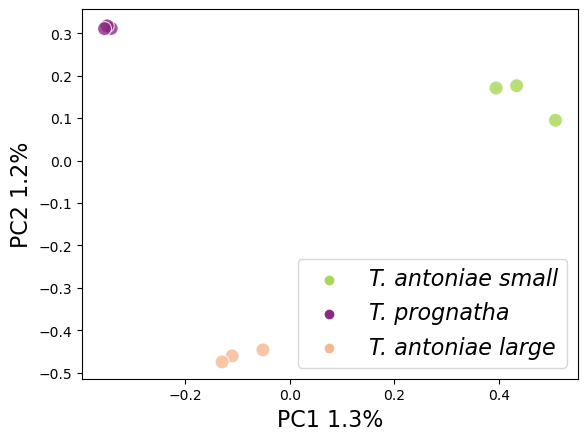


[Supplementary figure S8](#figur_PCA). **Visualisation of the first and second principal components** based on the SNP dataset for Lake Matano roundfin species.


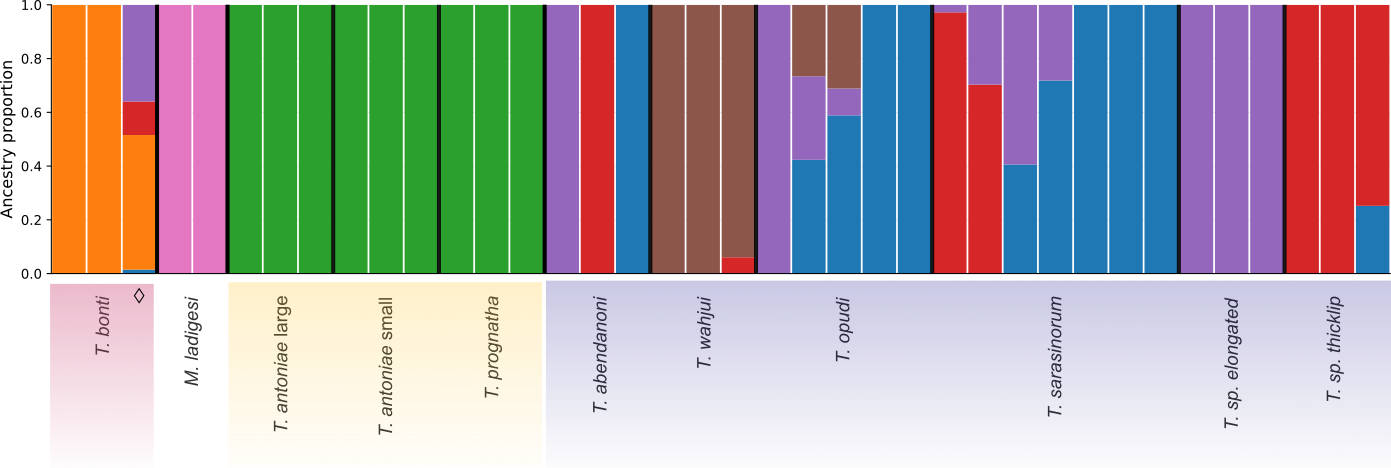


[Supplementary figure S9](#figur_admixture). **Ancestry composition based on admixture analysis.** Each bar is an individual, bars are coloured according to the proportion of ancestry assigned to each of the seven inferred genetic clusters. Diamond shape (
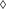
) indicates T. bonti 1, the presumed recent hybrid sample. Sharpfin species are highlighted in purple, roundfins in yellow and the T. bonti samples are indicated in red background shading.


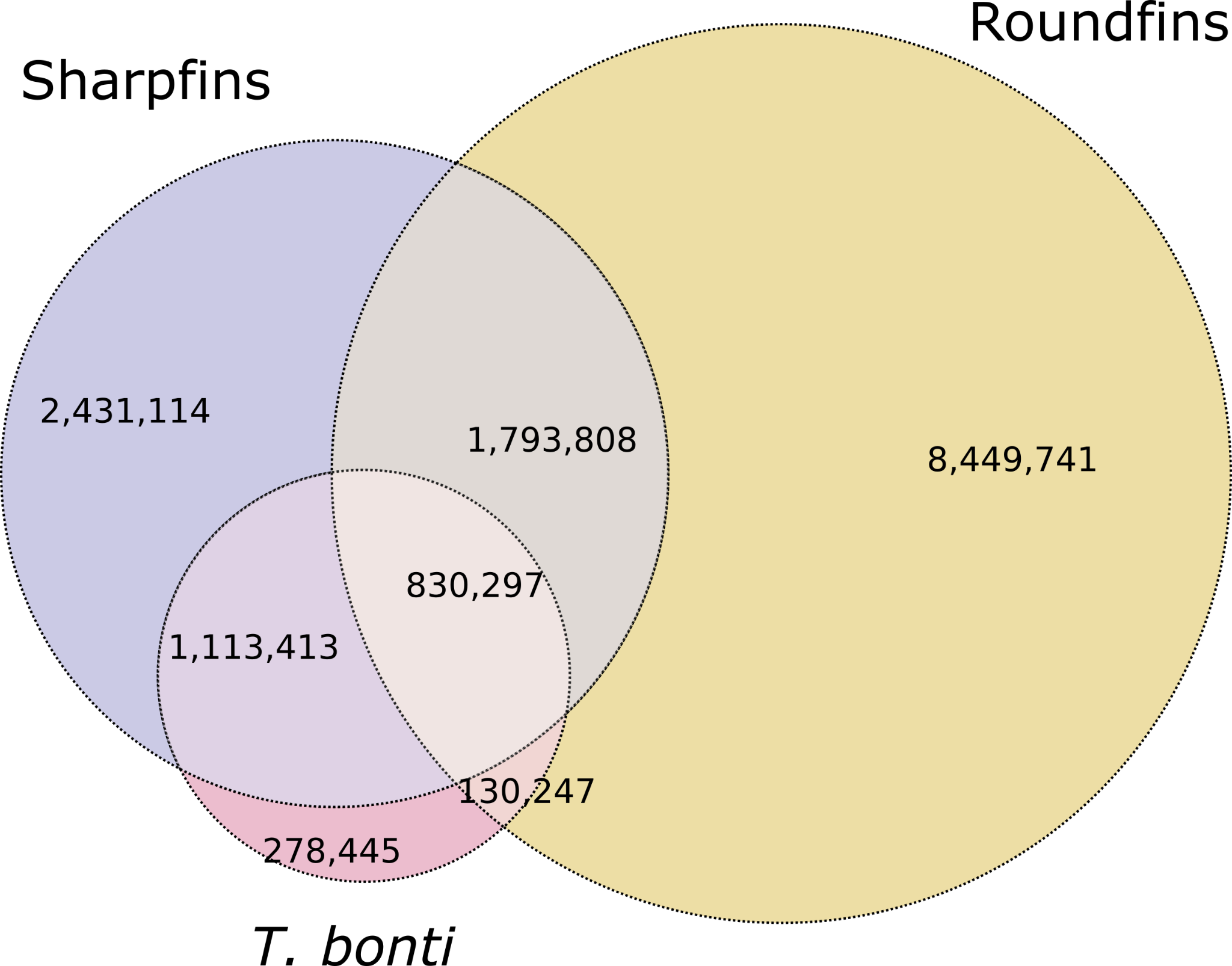


[Supplementary figure S10](#figur_venn). **Venn-diagram of shared and private variable sites** in sharpfins (n = 24), roundfins (n = 9) and *T. bonti* (n = 2)*.* Outer circles represent the number of variable sites unique to the corresponding group. Overlapping areas indicate the number of variable sites shared between the respective groups, i.e., where both alleles were present in both or all three groups.


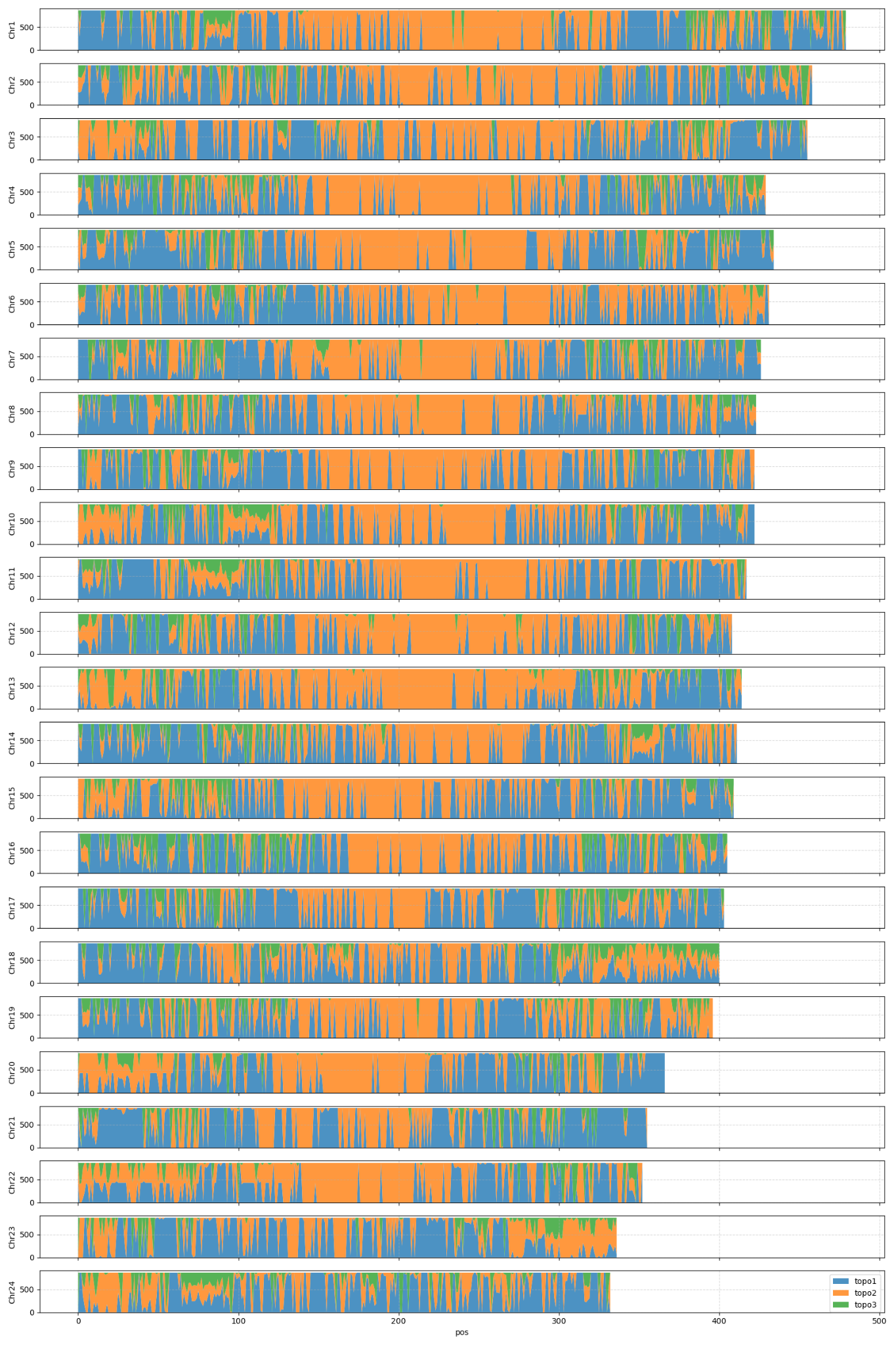


[Supplementary figure S11](#figur_twisst). **Twisst analysis showing frequency of topologies** in 100kb windows over the genome. Topo1, blue: (((sharpfins, roundfins), *T. bonti*), *M. ladigesi*); Topo2, orange: (((sharpfins, *T. bonti*), roundfins), *M. ladigesi*); Topo3, green: (((roundfins, *T. bonti*), sharpfins), *M. ladigesi)*. For each window the proportion of subsampled trees supporting each of the topologies is indicated.


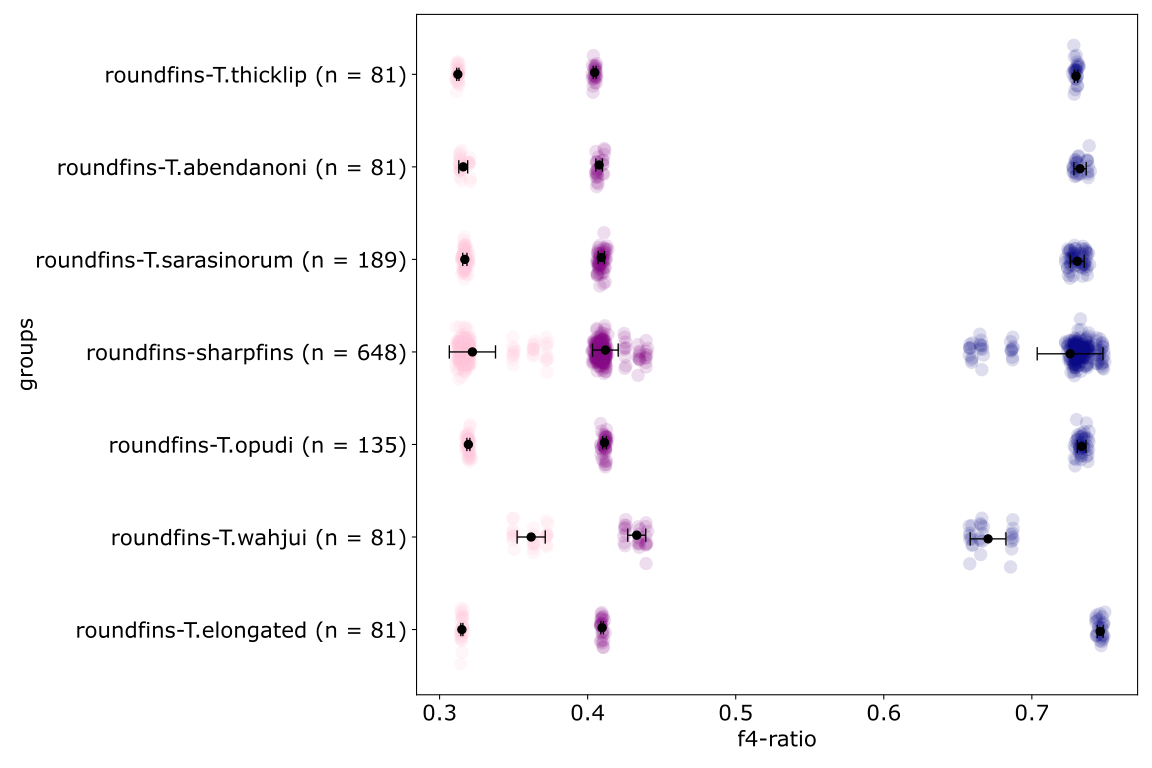


[Supplementary figure S12](#figur_f4). **Excess allele sharing (f4-ratio)** between *T. bonti* 3 (pink), *T. bonti* 2 (purple), *T. bonti* 1 (blue) (P3) and the sharpfin species indicated on Y axis (P2), relative to the roundfins (P1)

**
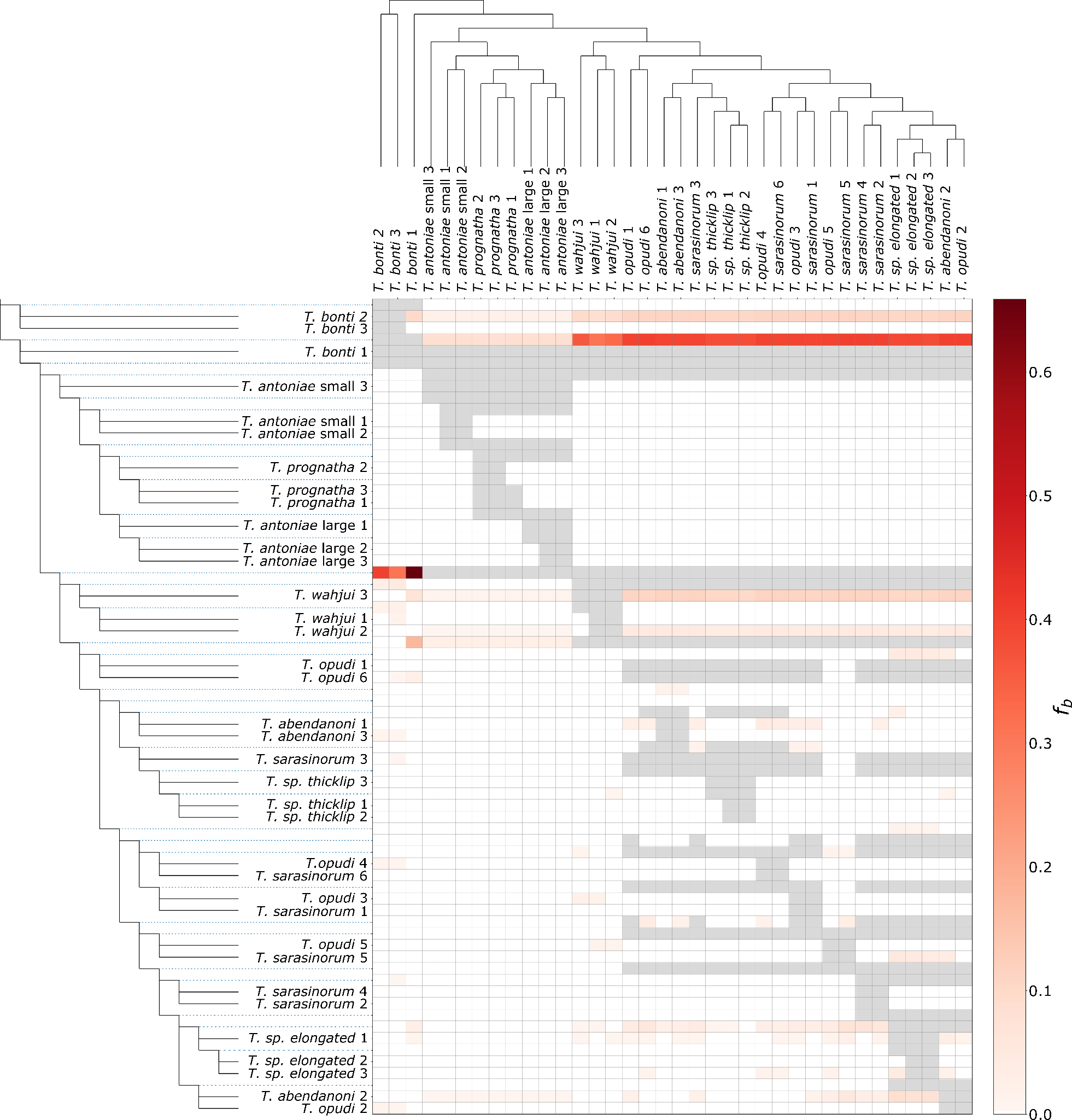
**

[Supplementary figure S13](#figur_fbranch). **Heatmap of pairwise f-branch (fb) statistic**, based on SNP dataset. Higher values (darker colour) indicate more excess allele sharing between specimens.

[Supplementary table S1](#table_samples). **Sample information** - sample ID as used in the manuscript and additional information about the samples used in the study. Sampling locations correspond to locations indicated in Supplementary figure S3.


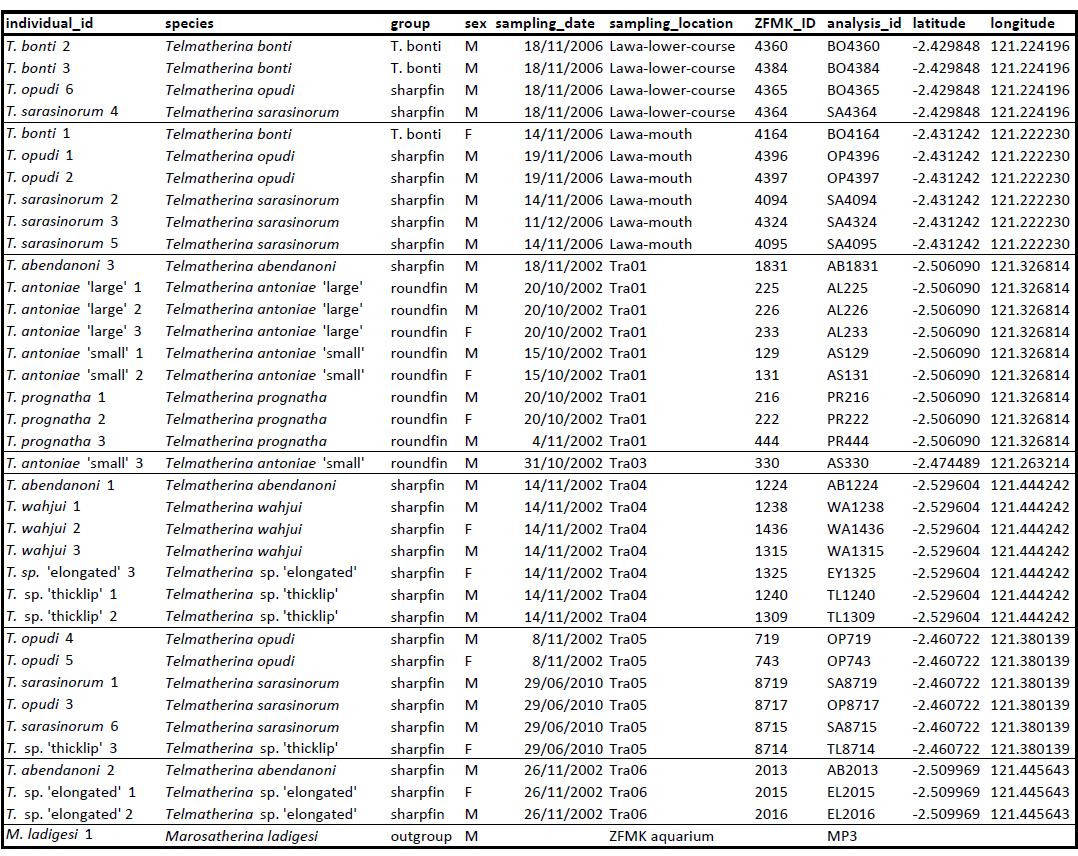


[Supplementary table S2](#table_chrom). **Chromosomes of fTelBon1:** chromosome size, GC-content in percentage, average coverage and accession number


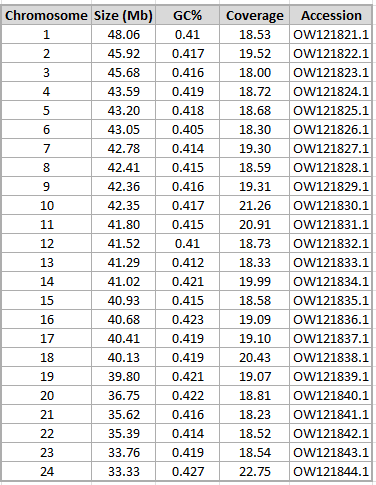

Supplement: Supplementary file 1 — Figure S1: Filter settings and number of sequences removed per filter. Area highlighted in red indicates the filter threshold (a) for the nuclear genome and (b) for the mitochondrial DNA. Figure S2: Assembly statistics for reference genome assembly of Telmatherina bonti . The BlobToolKit Snailplot shows N50 metrics and BUSCO gene completeness. The main plot is divided into 1000 size‐ordered bins around the circumference with each bin representing 0.1% of the 986,039,709 bp assembly. The distribution of sequence lengths is shown in dark grey (length measured from outside to inside) with the plot radius scaled to the longest sequence present in the assembly shown in red (48,058,054 bp). Orange and pale‐orange arcs show the N50 and N90 sequence lengths (41,523,244 and 35,388,860 bp), respectively. The pale grey spiral shows the cumulative sequence count on a log scale with white scale lines showing successive orders of magnitude. The blue and pale‐blue area around the outside of the plot shows the distribution of GC, AT and N percentages in the same bins as the inner plot. Top right: summary of complete, fragmented, duplicated and missing BUSCO genes in the actinopterygii_odb10 set. An interactive version of this figure is available at https://blobtoolkit.genomehubs.org/view/Telmatherina%20bonti/dataset/CAKOGG01/snail. Figure S3: Distribution plot of base coverage in ERR8978456 against position for sequences in assembly CAKOGG01. 100 kb windows are coloured by phylum. Figure S4: (a) Cumulative sequence length for assembly CAKOGG01. The grey line shows cumulative length for all sequences. Coloured lines show cumulative lengths of sequences assigned to each phylum using the buscogenes taxrules. An interactive version of this figure is available at https://blobtoolkit.genomehubs.org/view/Telmatherina%20bonti/dataset/CAKOGG01/cumulative. (b) Blob plot of base coverage in ERR8978456 against GC proportion for sequences in assembly CAKOGG01. Sequences are coloured by phylum. [file MEC-35-e70414-s001.docx]
